# Autoxidation Products of the Methanolic Extract of the Leaves of *Combretum micranthum* Exert Antiviral Activity against *Tomato Brown Rugose Fruit Virus* (ToBRFV)

**DOI:** 10.3390/molecules27030760

**Published:** 2022-01-24

**Authors:** Valeria Iobbi, Anna Paola Lanteri, Andrea Minuto, Valentina Santoro, Giuseppe Ferrea, Paola Fossa, Angela Bisio

**Affiliations:** 1 Department of Pharmacy, University of Genova, Viale Cembrano 4, 16148 Genova, Italy; valeria.iobbi@edu.unige.it (V.I.); paola.fossa@unige.it (P.F.); 2 CeRSAA—Centro di Sperimentazione e Assistenza Agricola, Regione Rollo 98, 17031 Albenga, Italy; labfito@cersaa.it (A.P.L.); andrea.minuto@rivlig.camcom.it (A.M.); 3 Department of Pharmacy, University of Salerno, Via Giovanni Paolo II 132, 84084 Salerno, Italy; vsantoro@unisa.it; 4 Azienda Sanitaria Locale 1, Regione Liguria, Via Aurelia 97, Bussana, 18038 Sanremo, Italy; giuseppeferrea@gmail.com

**Keywords:** ToBRFV, *Combretum micranthum*, catechinic acid, 4-hydroxybenzoic acid, coat protein, molecular docking

## Abstract

*Tomato brown rugose fruit virus* (ToBRFV) is a new damaging plant virus of great interest from both an economical and research point of view. ToBRFV is transmitted by contact, remains infective for months, and to-date, no resistant cultivars have been developed. Due to the relevance of this virus, new effective, sustainable, and operator-safe antiviral agents are needed. Thus, 4-hydroxybenzoic acid was identified as the main product of the alkaline autoxidation at high temperature of the methanolic extract of the leaves of *C. micranthum*, known for antiviral activity. The autoxidized extract and 4-hydroxybenzoic acid were assayed in in vitro experiments, in combination with a mechanical inoculation test of tomato plants. Catechinic acid, a common product of rearrangement of catechins in hot alkaline solution, was also tested. Degradation of the viral particles, evidenced by the absence of detectable ToBRFV RNA and the loss of virus infectivity, as a possible consequence of disassembly of the virus coat protein (CP), were shown. Homology modeling was then applied to prepare the protein model of ToBRFV CP, and its structure was optimized. Molecular docking simulation showed the interactions of the two compounds, with the amino acid residues responsible for CP-CP interactions. Catechinic acid showed the best binding energy value in comparison with ribavirin, an anti-tobamovirus agent.

## 1. Introduction

Phyto-viruses are one of the most common type of etiological agents of plant disease [1] and pose a major threat to agriculture worldwide [2,3]. Literature data report that some viruses can infect more than 1000 different plant species, comprising more than 85 families, and in subtropical and tropical regions, a viral infection can lead to a loss of up to 98% of the crop [3,4,5]. Tomato (*Solanum lycopersicum* L.) is one of the most widespread and economically important vegetable crops in the world. Italy is among the top ten tomato producers worldwide, with an annual production of about five million tons [6], and viruses drastically reduce the yield and quality of tomato crops [7]. *Tobamovirus*, the largest genus of the *Virgaviridae* family affecting tomato crops, represents a serious threat to the profitable production of this vegetable, due to ease of transmission, environmental stability, climate change, long-distance dispersal through offshore seed production, and global trade in seeds [8]. *Tobacco mosaic virus* (TMV) is one of the most important species of *Tobamovirus* infecting tomatoes, and it is widely used as a model to study host-pathogen interaction and virus evolution [9]. *Tomato brown rugose fruit virus* (ToBRFV) is a novel virus belonging to *Tobamovirus*, first reported in 2015 in Jordan [10] and afterwards in China [7], Germany [11], Israel [12], Italy [13], Mexico [14], Palestine [15], Turkey [16], United Kingdom [17], and United States [18], thus becoming a significant source of rising global outbreaks of *Tobamovirus*. ToBRFV is considered more virulent than other known tomato-infecting tobamoviruses. In addition to transmission via seeds, mechanical contacts, and stability in soil, clothing, various agricultural tools and glasshouse structures [19,20,21] such as other tobamoviruses, ToBRFV breaks Tm-1, Tm-2, and Tm-2^2^ resistance genes, which are present in many commercial tomato cultivars with disease incidence close to 100% in some fields [7]. So far, tomato cultivars resistant to ToBRFV hae not been reported [7]. Control measures against ToBRFV are limited and based on the elimination of infected plants and strict hygiene measures. Currently, used disinfection systems are not very effective and often potentially dangerous for the operator.

Chemical control measures damaging virus epidemics in cultivated plants faced with increasing concerns over sustainability, human health, environment protection and social issues [22]: pesticides are recognized as a central responsible factor for the observed terrestrial biodiversity declines [23], as they are found as contaminants in soil, air, water, and non-targeted organisms [24]. Plants extracts and pure compounds have been described as potential tools to manage different crop pests and weeds, due to easily available sources, low cost, biodegradability, varied modes of action, and low toxicity to non-targeted organisms [25,26,27,28,29]. Activity of plant compounds against phyto-viruses has been reported [30,31,32]. Plant phenolic compounds are involved in plant defense [33,34] and show several properties against a wide spectrum of plant pathogens [35,36], specifically against various viruses, including tobamoviruses [37,38,39,40].

The leaves of *Combretum micranthum* G.Don (Combretaceae) are widely used, in the traditional medicine of West Africa, as a tea for the treatment of various diseases [41,42]. The species contain polyphenolic compounds, including *O*-glycosylflavones, *C*-glycosylflavones, flavans [41,43,44], and flavan-piperidine alkaloids (kinkéloids) [42]. Additionally, other alkaloids, including stachydrine, choline, hydroxyl-stachydrine [45], *m*-inositol, and sorbitol, have been identified [46].

In 1993, a study conducted in our laboratory showed that the alkaline autoxidized methanolic extract of the leaves of *C. micranthum* inhibited the in vitro replication of HSV-1 and HSV-2 [47,48]. Based on the observation that a freshly prepared solution of the methanolic extract showed no antiviral activity, and a week-old one showed only a weak activity, while the methanolic extract heated under alkaline pH was active, the research group hypothesized that ageing, and specifically alkaline catalysis, combined with heating at temperatures above 90 °C, promoted the formation of active compounds from inactive precursors. To confirm this hypothesis, and considering that catechins, the main constituents of the methanolic extract, undergo rearrangement to catechinic acid (**1**) in hot alkaline solution [49], catechinic acid was prepared from pure (+)-catechin [49], then it was oxidized in alkaline oxygen saturated solution at high temperature, and the reaction mixture (AOCA) was tested. The almost comparable antiherpetic activity and the very similar IR and UV absorption curves of the alkaline oxidized methanolic extract and AOCA confirmed that the oxidation products of catechinic acid were mainly responsible for the antiviral activity in the oxidized extract, but their chemical structures were not defined.

Due to the antiviral activity of plant extracts containing catechins against TMV [50,51,52,53], aim of the present work was to investigate the antiviral activity of the alkaline oxidized methanolic extract of the leaves of *C. micranthum* against ToBRFV, and to identify its main constituents. In vitro experiment [54], combined with mechanical inoculation on test plants [55], were performed to evaluate the viral infectivity deactivation potential. The absence of detectable RNA genome of ToBRFV, after a short exposure to the selected extract and compounds, and the absence of viral genome in the host plant, as a possible consequence of disassembly of virus coat protein (CP), was considered indicative of viral infectivity deactivation [56,57].

**Figure 1:**
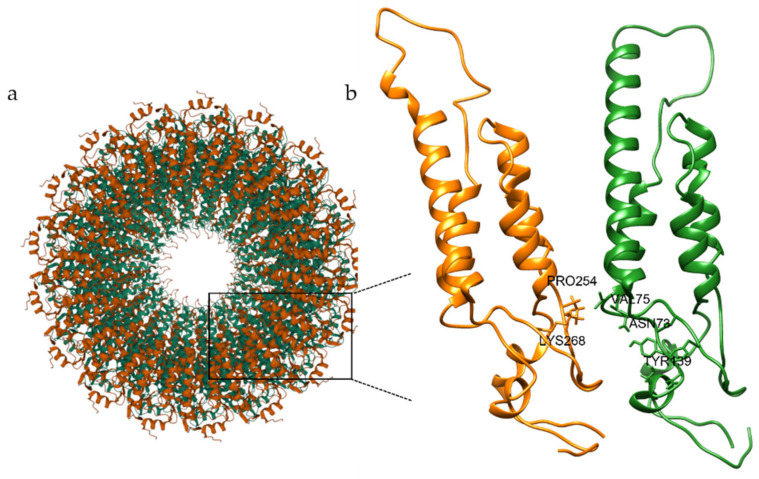
Crystal structure and active site of TMV CP. (**a**) The 20S disk is a four-layer cylindrical structure with 17 CP molecules in each ring [59,60]. (**b**) Architecture of the two CP subunits (chain A in green and chain B in orange) depicting the binding site residues in between them.

It is well known that the CPs of tobamoviruses are essential in maintaining and protecting the RNA genome [56,57]. TMV CP is characterized as a well-ordered dimer of a bilayered cylindrical disk, formed by 34 chemically identical subunits assembled around a single strand of viral RNA [58] (Figure 1).

**Figure 2:**
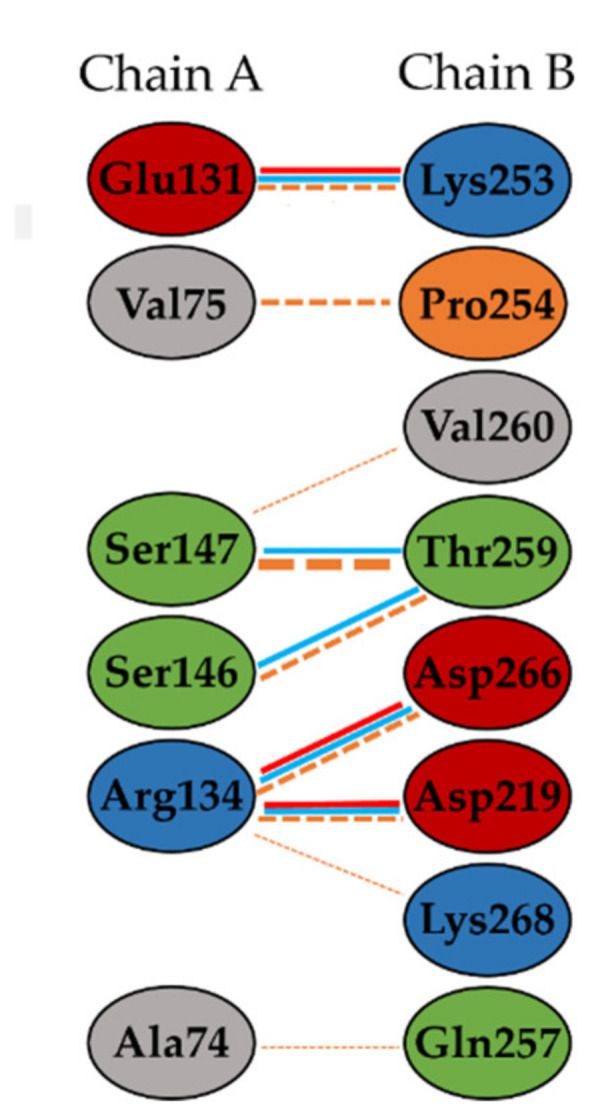
Interactions between chain A and chain B in TMV CP [74]. The protein residues are represented as follows: the negatively charged residues are indicated in red, positive residues are in cyan, neutral residues are shown in green; aliphatic residues are grey; Pro and Gly are orange. H-bonds are depicted as cyan lines; salt bonds are reported as red lines; non-bonded contacts are light orange dotted lines. The number of H-bond lines between any two residues indicates the number of potential hydrogen bonds between them. The width of the striped line of non-bonded contacts is proportional to the number of atomic contacts.

CPs show interesting biological functions, playing a key role in the encapsulation and protection of single strand RNA virus genome [56,61,62], and being involved in the symptom modulation [63,64,65]. CPs are involved in the CP mediated resistance (CP-MR), a phenomenon in which mutant also reduced the formation of virus replication complexes [66,67]. Mutational studies of TMV CP demonstrated the importance of the inter-subunit interactions to stabilize the virion and prevent virus disassembly [66,68]. These interactions are mainly due to Ala74, Val75, Arg134, Ser146, and Ser147 residues [59]. In addition, Tyr139 is involved in various hydrophobic interactions around the rings of disk assembly [69,70]. The Arg113-Arg90 interaction has also been observed [59]. These residues have been reported to play a significant role in the formation of TMV particles responsible for the disease promotion and to be involved in the disassembly of viral particles [56,57,59,69,71]. Therefore, it has been suggested that alterations affecting the stability of CP structures influence recognition by the host [61,72]. Phenolic compounds may inhibit the assembly of the CP by blocking the interaction between chain A and chain B (Figure 2), interrupting the CP-CP interactions [73].

The genome organization of ToBRFV has been described as typical of *Tobamovirus* and therefore related to TMV [7]. Phylogenetic analysis demonstrated that ToBRFV could be originated from a common ancestor of *Tomato mosaic virus* (ToMV) and TMV [7,75,76]. Unfortunately, the three-dimensional structure of ToBRFV CP has not yet been resolved. Due to the high percentage of sequence identity between TMV CP and ToBRFV CP, we built a ToBRFV CP homology model using the TMV CP 3D structure as a template [59]. Molecular docking studies were then performed on the homology model, aiming to investigate and compare the binding energies and the molecular interactions performed by the studied compounds at the active site of the virus CP.

## 2. Results

### 2.1. Chemical Analysis of the Autoxidized Methanolic Extract of the Leaves of C. micranthum

The methanolic extract of the leaves of *C. micranthum* was submitted to alkaline oxidation, under heating at 100 °C, to obtain the alkaline autoxidized methanolic extract (AME) [47]. The semi-preparative HPLC purification of AME afforded 4-hydroxybenzoic acid (**2**) as main compound (Figure S1, Supplementary Materials), identified by the comparison of the spectroscopic data with those reported in the literature [77,78].

To verify that 4-hydroxybenzoic acid (**2**) derived from the alkaline oxidation at high temperature of catechin constituents of the methanolic extract, catechinic acid was subjected to the same alkaline oxidation [79]. For this purpose, synthetic catechinic acid (**1**) was obtained by the stereoselective transformation under alkaline conditions of (+)-catechin, following the method reported by Sears et al. [49], and identified by the comparison of the spectroscopic data with those reported in the literature [80]. Subsequently, **1** was subjected to alkaline oxidation at high temperature, following the method previously reported [47]. The semi-preparative HPLC purification of the alkaline autoxidation product of **1** (AOCA) afforded **2**, thus confirming the result obtained from the HPLC purification of AME.

### 2.2. Antiviral Activity Assay and Evaluation of Final Viability of the Virus

The antiviral activity of both AME and 4-hydroxybenzoic acid (**2**) was evaluated. Catechinic acid (**1**) was tested too. Since **2** was the main constituent of AME, both AME and **2** were tested at the same concentration. Catechinic acid (**1**) was evaluated at the same conditions. The presence of detectable RNA genome of ToBRFV, after a short exposure to the treatments in a multiwell plate, was checked with molecular detection of different encoding regions of the virus (Figure S11, Supplementary Materials). Swabs used for the sampling of inoculated but not treated wells (positive control) were positive for the presence of the virus. On the contrary swabs used for the sampling of inoculated and treated wells (**1**, AME, **2**, NaOCl), as well as swabs used for the sampling of not inoculated and not treated wells (negative control), were negative for the presence of the virus. Results obtained during the three trials, by the molecular detection of ToBRFV on the swabs used for the sampling of the wells, are summarized in Table 1.

**Figure 3:**
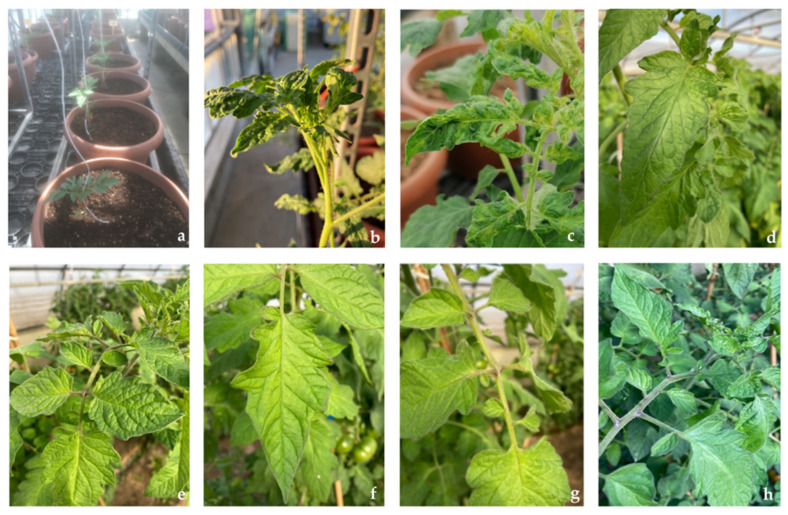
Plant inoculation test. (**a**): trial’s layout. (**b**–**h**): 35 days after the inoculation. (**b**,**c**): symptomatic apical leaves (plants inoculated with Positive Control); (**d**): not symptomatic apical leaves (plants inoculated with Negative Control); (**e**): not symptomatic apical leaves (plants inoculated with inoculum treated with **1**); (**f**): not symptomatic apical leaves (plants inoculated with inoculum treated with AME); (**g**): not symptomatic apical leaves (plants inoculated with inoculum treated with **2**); (**h**): not symptomatic apical leaves (plants inoculated with inoculum treated with NaClO).

After the antiviral activity assay, treated inoculum was used for mechanical inoculation of test plants to check the final viability of the virus (Figure 3a). Plants inoculated with swabs used for the sampling of inoculated but not treated wells (positive control) developed symptoms (blistering, distortion, and narrowing of leaves) starting from 21 days after the inoculation, during all three trials. Molecular analysis performed on the new apical leaves of the plants inoculated with positive control was positive for the presence of the virus 35 days after the inoculation (Figure 3b,c). On the contrary, plants inoculated with swabs used for the sampling of inoculated and treated wells (**1**, AME, **2**, NaOCl), as well as plants inoculated with swabs used for the sampling of not inoculated and not treated wells (negative control), did not develop symptoms during all three trials. Molecular analysis performed on the new apical leaves, 35 days after the inoculation, was always negative for the presence of the virus (Figure 3d–h).

Results obtained during the three trials, by the molecular detection of ToBRFV on the new apical leaves of the inoculated plants collected 35 days after the inoculation, are reported in Table 2. The young apical leaves of the 3 replicates were collected and analyzed together.

### 2.3. Homology Modelling and Validation

**Figure 4:**
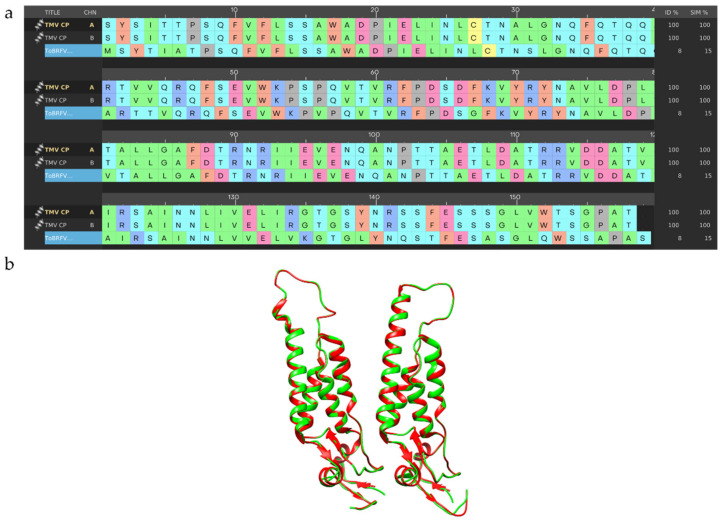
Sequence alignment of CP sequences and homology model of ToBRFV CP; (**a**) Sequences alignment between ToBRFV CP and TMV CP; (**b**) 3D comparison between ToBRFV CP homology model (green) and TMV CP crystal structure (red).

In the absence of a 3D model for ToBRFV CP, a homology modeling strategy was applied. Performing BLAST-P alignments [82] and Uniprot [83] searches, the TMV CP (P69687 CAPSD_TMV) sequence emerged as the most closely related to ToBRFV CP sequence (A0A0S2SZX3), with 89.3% of sequence identity (e-value: 6.1 × 10^−98^). The X-ray crystal structure of TMV CP with the best resolution (2.45 Å; PDB code: 1ei7) [59] was retrieved from the Protein Data Bank [84,85] and selected as a template. ToBRFV CP sequence differs from TMV CP sequence for 14 residues (Figure 4), among which Lys134A and Val255B are part of the CP active site. Considering the architecture of the monomers in the template structure, to retain the three-dimensionality of the structure, the 14 mutations were edited manually. Protein preparation and refinement tool [86] allowed us to align the two 3D-structures, obtaining an RMSD of 0.193 Å.

**Figure 5:**
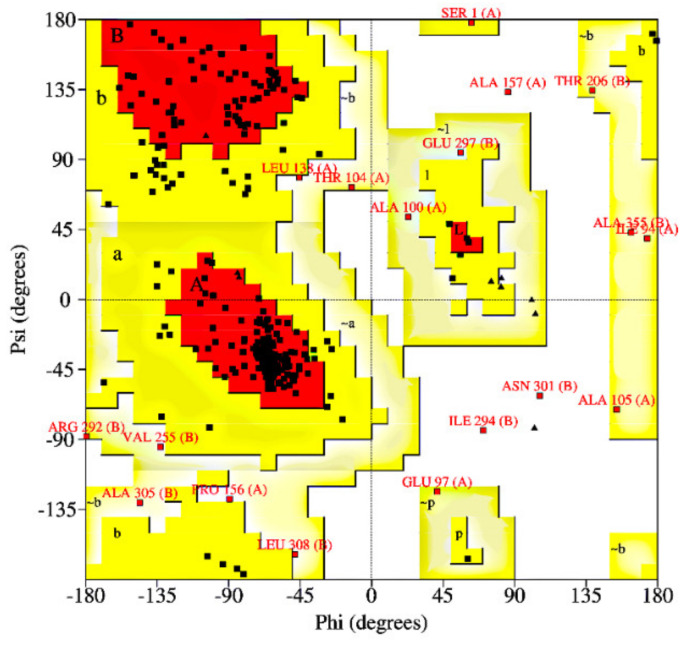
The Ramachandran plot of ToBRFV CP homology model. There are 218 residues located in the most favored region (red), 51 residues in the additional allowable region (yellow), 14 residues in the generous allowable region (light yellow), and only 3 residues in the prohibited region (white). Gly is plotted as triangles, Pro is plotted as squares, and all other residues are plotted as circles.

The assessment of the Ramachandran plot, produced by PROCHECK [87] (Figure 5), showed 76.2% residues in the most popular regions, 17.8% residues were found in the allowed areas, and 5.9% residues were found outside. Therefore, since most residues were found in a favorable region, while only few residues were found in the external region, we could conclude that the quality of the model was good.

### 2.4. Molecular Docking Studies

**Figure 6:**
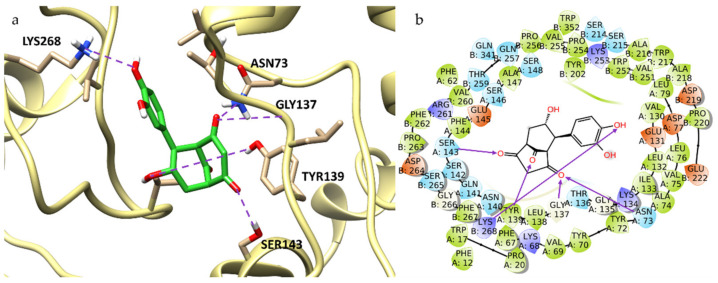
Binding pose (**a**) and interactions (**b**) of catechinic acid at the ToBRFV CP active site. (**a**): the protein is reported as light-yellow ribbons; catechinic acid is reported as green capped sticks. H-bonds are presented as purple dotted lines. (**b**): catechinic acid is surrounded by the protein residues represented as follows: the negatively charged residues are indicated in red, polar residues are in cyan, hydrophobic residues are shown in green, and H-bonds are depicted as purple arrows.

We evaluated the binding of catechinic acid (**1**) and 4-hydroxybenzoic acid (**2**) with ToBRFV CP, using the Schrödinger Suite 2021-4 [88], and focusing the attention on the region between the CP subunits, which mainly include residues Asn73, Ala74, Val75, Lys134, Thr136, Gly137, Tyr139, Ser143, Asp219, Glu222, Val251, Lys253, Val255, Val260, and Lys268, all described as important for TMV by several authors [73,89,90,91]. In such a defined region, catechinic acid complex was stabilized by several H-bonding interactions with Asn73A, Gly137A, Tyr139A, Ser143A, and Lys268B and by a hydrophobic interaction with Val260B (Figure 6). Additionally, 4-hydroxybenzoic acid was bound to different key residues, involved in direct interaction between the two CP-CP subunits. According to our calculations, the ligand hydroxyl group made four H-bonds with Asp219B, Glu222B, Lys253B, Pro254B; the carboxylic group bound Lys268B with an H-bond, and 4-hydroxybenzoic acid was also involved in hydrophobic interaction with Asp219B, Lys253B, and Val255B. This predicted binding mode was further stabilized by a salt bridge between the carboxylic group and Lys268. Lys268B, according to our calculations, was also engaged in a π-cation interaction with the ligand aromatic ring (Figure S12, Supplementary Materials). Thus, catechinic acid and 4-hydroxybenzoic acid acquired a stable conformation inside the binding pocket, interacting with a high number of residues. Ribavirin is described in literature as a tobamovirus inhibitor, specifically active against TMV [92,93]. Several authors recently investigated the interaction pattern of ribavirin-CP complex and its binding between two CP subunits [73,89,91,94,95,96,97]. On these premises, we found it interesting to investigate its molecular interaction with our homology model, using it as a template in docking studies. Ribavirin emerged as strongly bound in the active site of the protein by six H-bonds formed by its hydroxyl groups of β-D-ribofuranosyl moiety as well as Asn73A, Gly137A, Ser143A, and Leu138A residues. Additional interactions were made by Lys134 and Lys268B with the oxygen atom of the carboxamide group. The ribavirin amino group interacted with Pro254B. All these amino acids are considered key residues in the target (Figure S13, Supplementary Materials).

Extra precision docking mode confirmed the results obtained with the standard procedure (Material and Methods, 4.8.3). Catechinic acid achieved the best binding energy value (−38.794 kcal/mol), followed by ribavirin (−35.285 kcal/mol) and 4-hydroxybenzoic acid (−25.688 kcal/mol). Ligand-binding energies and interactions are listed in Table 3.

## 3. Discussion

In a previous work, the antiviral activity of the methanolic extract of the leaves of *C. micranthum* after alkaline oxidation at high temperature (AME), as mediated by autoxidation products of catechin and catechinic acid (**1**), was reported [47,79,80,98,99,100]. In the present work, the chemical investigation of AME led to the isolation of **2** as the main compound, characterized as the 4-hydroxy derivative of benzoic acid, commonly produced from thermolytic degradation of flavonoids [101,102]. The other compounds described by Ohara et al. [100] and Hashida et al. [80], as originated by the alkaline degradation of catechinic acid at lower reaction temperatures, were not detected. To confirm that **2** was really the main product of degradation of the polyphenolic constituents of the methanolic extract of *C. micranthum*, and considering that catechins produce catechinic acid in hot caustic solutions [79], synthetic catechinic acid (**1**) was prepared and subjected to the same alkaline degradation as the methanolic extract. The HPLC purification of the obtained product afforded **2**.

AME, **2**, and **1** were then evaluated for their antiviral activity. The presence of detectable RNA genome of ToBRFV on the inoculated surface, after a short exposure to the treatments, was checked with molecular detection of different encoding regions of the virus. The treated inoculum was then used for mechanical inoculation of test plants to check the final viability of the virus. Molecular analysis was performed on inoculated plants to verify the presence of the RNA genome of ToBRFV on the new apical leaves. The in vitro test, performed to evaluate the antiviral activity of AME, **2**, and **1** showed their effectiveness against ToBRFV when applied as solutions at the concentration of 32 ppm on a contaminated surface for an exposure time of at least 60 s. The loss of viability of the inoculum, in the conditions of the test, was confirmed by the absence of symptoms on the inoculated test plants during all the three trials. The molecular analysis, performed on the same test plants 35 days after the inoculation, confirmed the absence of virus RNA genome inside the plants. Strong oxidizing agents such as sodium hypochlorite are considered the most effective disinfectants against the small non-enveloped viruses, such as noroviruses [103]. The results obtained with AME, **2**, and **1** were comparable with those obtained with the solution of sodium hypochlorite, a commercially available virucidal sanitizing agent, in the same experimental conditions. The activity of **2,** the main constituent of AME, was superimposable to AME. Catechinic acid (**1**) showed the same activity.

*Tobacco mosaic virus* (TMV) has been widely used as a model of tobamoviruses to investigate viral evolution and virulence [9] and as a representative virus in the study of new antiviral agents [38]. Phylogenetic studies reported ToBRFV to be closely related to tobamoviruses, such as TMV [7]. In recent years, other authors considered TMV CP an important target for screening viral agents against tobamoviruses [90,97,104,105,106,107]. Careful literature overview highlighted several in silico studies based on the crystal structure of TMV CP (PDB code 1ei7) [39,72,73,89,91,94,97,105,106,107]. Due to the high conservation of the tobamovirus CP sequences [108] in this study, we considered the TMV CP sequence as a template. Several TMV CP structures are available in the PDB [84,85], with a resolution spanning from 2.8 Å to 2.4 Å [59,69]. Among these, we selected the best solved one, 1eI7 (2.45 Å) [59]. The high-resolution refined atomic model of the four-layer aggregate of the TMV CP, as a dimer of a bilayered cylindrical disk formed by 34 chemically identical subunits assembled in a right-handed helix around a single strand of viral RNA, is reported in the literature [59,109]. The predominant species at pH 7.0 is the ‘20S’ aggregate [56]. The subunits are highly ordered, and they interact with each other, both laterally and axially, via salt bridges and H-bonds [59]. These interactions between viral CPs are involved at every stage of a viral infection [58]. The aggregation of CP, initiated by RNA recognition, plays an important role in viral assembly, initiation, and elongation [57,110]. Blocking the interactions between chain A and chain B, and thus interrupting the CP-CP interaction is considered one of the strategies for the development of antiviral agents [73]. The formation of a complex protein-ligand generates a reorganization in the target structure that affects the physiology of the protein itself. All reported inhibitors are positioned between the two subunits of CP that protect the single strand RNA [73]. Occupation of this active site, using small molecule inhibitors, produces an alteration in interactions between the two subunits of CP [72,106,111], which impacts CP structural stability.

With the aim of supporting the experimental data obtained in vitro, we investigated the molecular interactions of catechinic acid (**1**) and 4-hydroxybenzoic acid (**2**) with ToBRFV CP active site. In this docking study, the two compounds demonstrated an interesting interaction with the target and a positive alignment with ribavirin, the most commercialized and extensively used TMV chemical inhibitor [94,112]. Additionally, **1** and **2** were found to fit well with ToBRFV CP, binding the amino acid residues (Asn73, Ala74, Val75, Lys134, Thr136, Gly137, Tyr139, Ser143, Lys253, Val255, Val260, and Lys268), which are responsible for CP-CP interactions. Ligand interactions also involved the hydroxyl groups of both catechinic acid and 4-hydroxybenzoic acid, in full agreement with the interactions previously reported in the literature [39,73,89]. It could be supposed that the ligand binding between the two protein subunits may influence the interactions between them. The interactions of catechinic acid and 4-hydroxybenzoic acid may, therefore, generate modification in the CP protein stability and functioning, thus obstacling virus particles self-assembly. Catechinic acid showed a better binding energy than ribavirin, interacting efficiently with the virus CP active site and performing better than the already used inhibitor. These results suggest the hypothesis of a potential antiviral activity of catechinic acid on ToBRFV CP and support the idea that natural plant compounds can interfere with virulence progress.

Several studies reported synthetic or semi-synthetic compounds showing a good inactivating activity against TMV, superior to that of ribavirin [73,91,94,95,96]. Recent research highlighted several natural anti-TMV metabolites, extracted from plants: the seeds of *Sophora tonkinensis* [40], *Chelidonium majus* [112], *Tithonia diversifolia* [113], and *Picrasma quassioides* Benn. [114], *Cnidium monnieri* [115], and essential oils isolated from Chinese indigenous aromatic plants [116]. A review by Islam et al. [117] summarizes the status of the research on the topic. The literature survey did not report papers about plant compounds active against ToBRFV, and to the best of our knowledge, this study represents the first report on this topic. Finally, our investigation highlights catechinic acid as a promising agent against phyto-viruses.

## 4. Materials and Methods

### 4.1. Chemicals

(+)-Catechin analytical standard Sigma-Aldrich^®^ was purchased from Merck (Darmstadt, Germany). All reagents were of analytical grade and purchased from Merck (Darmstadt, Germany). Deionized water was purified by Milli-Q^®^ plus system (Merk Millipore, Bedford, MA, USA).

### 4.2. General Experimental Procedures

NMR experiments were performed on a Bruker DRX-600 spectrometer (Bruker Bio-Spin GmBH, Rheinstetten, Germany) equipped with a Bruker 5 mm TCI CryoProbe at 300 K and a Bruker DRX-400 spectrometer. All 2D NMR spectra were acquired in CD_3_OD, and standard pulse sequences and phase cycling were used for COSY, HSQC, and HMBC spectra. The NMR data were processed using UXNMR software. HRESIMS data were acquired in the positive ion mode by an LTQ Orbitrap XL mass spectrometer (Thermo Fisher Scientific, San Jose, CA, USA). Semi-preparative HPLC was carried out using a Waters W600 pump equipped with a Rheodyne Delta 600 injector, a 2414 refractive index detector, and a 2998 photodiode array detector (all Waters Corporation, Milford, MA, USA). A C18 column, SymmetryPrep C18, 7.8 × 300 mm ID, 7 µm particle size (Waters) was used, at room temperature, flow rate 2.0 mL/min, sample loop 100 µL, eluents A: H_2_O, B: CH_3_OH, gradient: 25% to 30% B in 50 min, then 30% B to 35% in 40 min. The column was equilibrated with 75% A for 20 min prior to each analysis. The effluent was monitored at 280 nm.

### 4.3. Plant Material

Dried leaves of C. micranthum (Kinkeliba leaves) were obtained from a commercially available source. A voucher specimen (82/19/interno) was deposited in the Laboratorio Fitopatologico, CERSA, Albenga (SV) Italy.

### 4.4. Preparation of Alkaline Autoxidized Methanolic Extract and Purification

The alkaline autoxidized methanolic extract (AME) of the leaves of C. micranthum was prepared, as previously reported [47]. Briefly, dried leaves of C. micranthum (300 g) were extracted with 95% methanol. After evaporation of most of the solvent, the residue (100 mL) was washed with ethyl acetate (300 mL) and concentrated under reduced pressure to dryness (60 g). The methanolic extract (3 g) was refluxed for 45 min under N_2_ in a solution of NaOH (2.4 g in 180 mL of water). Air was subsequently bubbled through the solution while the flask was immersed in a water bath at 100 °C. After 1.5 h, the brown solution was cooled, passed through Amberlite IR-120 (H^+^ form, 35 mL) (Merck, Darmstadt, Germany) ion exchange resin column, adjusted to pH 7, and evaporated to dryness (1.7 g). AME (200 mg) was then purified by semi-preparative HPLC affording **2** (20.0 mg, t_R_ 27 min),

4-hydroxybenzoic acid (**2**). ^1^H NMR (600 MHz, CD_3_OD): δ = 7.87 (d, 2H, *J* = 8.00 Hz, CH (2/6)), 6.82 (d, 2H, *J* = 8.01 Hz, CH (3/5)) ppm. ^13^C NMR (150 MHz, CD_3_OD): δ = 170.2 (COOH), 163.4 (COH, C4), 133.0 (CH, C2/6), 122.7 (C, C1), 116.2 (CH, C3/5) ppm (assignments were confirmed by HSQC and HMBC experiments). HRESIMS *m/z* 137.0249 [M-H]^+^ (calcd. for C_7_H_6_O_3_, 137.0244). (Figures S2–S5, Supplementary Materials).

### 4.5. Preparation of Catechinic Acid and of Its Alkaline Autoxidation Product

Catechinic acid (**1**) was prepared following the method described by Sears et al. [49] by adding (+)-catechin (4.5 g) to a refluxing solution of 0.5% NaOH with a continual N_2_ flush for 45 min, cooling in ice, treating with Amberlite IR-120 resin (H^+^ form, 35 mL) (Merck, Darmstadt, Germany), and stirring for 1 h. The resin was then filtered off, and the solution evaporated to dryness to give a light brown resinous product, which was then purified by precipitation from acetone (200 mL). The precipitate was filtered to give 2.5 g of catechinic acid-acetone complex. Evaporation of the acetone mother liquor gave unsolvated catechinic acid (1.4 g). Then, 15 mg of catechinic acid was purified by semi-preparative HPLC affording **1** (10 mg, t_R_ 33 min).

Catechinic acid was then subjected to alkaline autoxidation, as previously described [47]. Briefly, air was bubbled through an alkaline solution (1.3 g NaOH in 20 mL of water) of **1** (1.00 g) for 30 min at room temperature, then for another 30 min at 55 °C, and finally, for 1.5 h at 100 °C. After cooling, the solution was passed through Amberlite IR-120 (H^+^ form, 35 mL) (Merck, Darmstadt, Germany), ion exchange resin column, adjusted to pH 7, and evaporated, in vacuum, to dryness (0.8 g) (AOCA). Then, 20 mg of AOCA was purified by semi-preparative HPLC, affording **2** (15.0 mg, t_R_ 27 min).

Catechinic acid (**1**). ^1^H NMR (600 MHz, CD_3_OD): δ = 6.68 ^a^ (1H, CH (2′)), 6.67 ^a^ (1H, CH (5′)), 6.55 (d, *J* = 8.2 Hz, 1H, CH (6′)), 5.40 (br s 1H, CH (3)), 4.47 (ddd, *J* = 10.9, 10.9, 5.4 Hz, 1H, CH (7)), 3.03 (dd, *J* = 4.5 Hz, 1H, CH (5)), 2.95 (dd, *J* = 4.2, 3.1 Hz, 1H, CH (1)), 2.83 (dd, *J* = 10.8, 4.5 Hz, 1H, CH(6)), 2.53 (ddd, *J* = 12.9, 5.4, 3.1 Hz, 1H, CH_2_ (8)), 1.77 (ddd, *J* = 12.9, 10.9, 4.2 Hz, 1H, CH2 (8)) ppm. ^13^C NMR (150 MHz, CD_3_OD): δ = 209.1 (C=O, C9), 191.3 (C-O, C2), 189.1 (COH, C4), 144.6 (COH, C3′), 144.5 (COH, C4′), 130.6 (C, C1′), 119.8 (CH, C6′), 115.4 (CH, C5′), 115.3 (CH, C2′), 104.9 (CH, C3), 66.7 (CHOH, C7), 66.4 (CH, C5), 58.2 (CH, C1), 37.0 (CH_2_, C8), ppm (^a^ = overlapped signals; assignments were confirmed by COSY, HSQC and HMBC experiments). (Figures S6–S9, Supplementary Materials).

### 4.6. Antiviral Activity Assay

The antiviral activity of **1**, AME, and **2** was evaluated by in vitro test (repeated three times) (Figure S11, Supplementary Materials). This assay was intended to define the effectiveness of these three compounds/extracts in degrading the RNA of the viral agent ToBRFV on a contaminated surface.

Based on the data available about the infectivity of Tobamovirus [118,119] and the lasting persistence of ToBRFV on contaminated surfaces [8,120], the laboratory previously validated a storage procedure for ToBRFV through the use of environmental swabs for the transfer and the survival of the virus for a period of at least 15 days (unpublished method). This procedure allowed the inoculum to be handled without the transfer of infected plant material, as well as to transfer the virus to a healthy plant to verify the infectivity of the pathogen through the use of the infected swab.

The well’s flat bottom surface of a cell culture multiwell plate (Greiner Bio-One GmbH, Frickenhausen, Germany) was inoculated with 0.1 mL of COPAN SRK^®^ (Copan Diagnostics, Inc. Murrieta, CA, USA) modified universal neutralizing solution, previously infected with ToBRFV. The inoculum was collected in Sicily during April 2020, three days before the plate inoculation, from the inner surface of a presumptive infected tomato harvest box by using a cotton swab dipped in 10.0 mL of COPAN SRK^®^ modified universal neutralizing solution, following the manufacturer’s instructions for surface sampling. Briefly, the pre-moistened swab was used for the sample uptake from an area of 25 cm^2^ and then maintained inside the tube with the solution until the analysis. The swab solution and the cotton swab were analyzed 2 days after sampling immediately upon arrival in the laboratory, to confirm the presence of ToBRFV.

Taking into account the order of magnitude of the application rate of commercial active ingredients usually applied for surface disinfection in agricultural systems [121], a preliminary test was set up to define the minimum concentration of AME, **2**, and **1** when RNA degradation could occur. Five different concentrations were tested. One minute after the plate inoculation, each well was treated with 0.1 mL of solution obtained dissolving AME, **2**, and **1**, respectively, in DNase/RNase free distilled water (UltraPureTM Invitrogen, Thermo Fisher Scientific Inc., Waltham, MA, USA), at the concentration of 8000, 1600, 320, 64, 12.8 ppm, respectively, to reach a final concentration of 4000 ppm, 800 ppm, 160 ppm, 32 ppm and 6.4 ppm, respectively. A solution of 2% NaOCl was used as a reference product. Furthermore, inoculated wells, treated only with 0.1 mL DNase/RNase free distilled water, were used as positive controls. Non-inoculated wells, treated with 0.1 mL clean modified universal neutralizing solution and 0.1 mL DNase/RNase free distilled water, were used as negative controls. Treated wells and controls have been prepared in triplicate. The plate was then maintained at room temperature with gentle agitation, and, after an exposure time of 60 s, the liquid was removed from the wells and a new cotton swab was used for the sampling of each well’s flat bottom surface. Considering the area of the well’s bottom (2.27 cm^2^), the liquid inside the tube was adjusted to 0.9 mL. Immediately after sampling, the tubes containing the cotton swabs and 0.9 mL of modified universal neutralizing solution were vortexed and then analyzed for the molecular detection of ToBRFV. Under the test conditions, the minimum concentration when RNA degradation occurred was 32 ppm for all the products (Table 1, Supplementary Materials). This concentration was then selected, and the antiviral activity assay was performed, as described above, and repeated three times.

### 4.7. Plant Inoculation

The viability of the pathogen, collected with the swab used for the inoculation and with the swabs used in the treated wells and controls (not treated wells), was verified by a biological assay inoculating three different healthy tomato plants per swab. For the mechanical inoculation, a proper quantity of carborundum (Merck KGaA, Darmstadt, Germany) as an abrasive was added to the swab tubes (1 g for tubes containing 10 mL solution and 0.1 g for tubes containing 0.9 mL solution). The inoculation was then carried out by dipping the cotton swab in the tube containing solution and abrasive and by rubbing the cotton swab briefly onto the upper surface of the leaves of 3 tomato plants [55]. The plants used for the inoculations were at BBCH (Biologische Bundesanstalt Bundessortenamt Chemische Industrie) [122] scale growth stage 22 (second primary apical side shoot visible), and they were previously analyzed to verify the absence of ToBRFV. Inoculated test plants were maintained in a containment greenhouse for 5 weeks and a symptom observation was conducted to assess the viral symptoms expression at 10, 21, and 35 days after the inoculation. During the last assessment, young apical leaves were collected, and molecular analysis was performed to confirm the presence of ToBRFV. The experiment was repeated three times. Inoculation tests were carried out following each in vitro experiment.

### 4.8. RT-PCR for the Detection of the Virus

The presence of ToBRFV in the swabs used for the inoculation of the wells and in the swabs used for the sampling of treated and not treated wells was verified by molecular analysis. Furthermore, after the last assessment of the inoculation test, samples composed by 6 leaves obtained from each group of 3 replicated plants (plants inoculated with the same swab) were analyzed for the presence of ToBRFV. Total RNA was extracted with RNeasy Plant Mini Kit (Qiagen GmbH, Hilden, Germany) from each swab solution, from the cotton swab, and from plant material following the manufacturer’s instructions. The RT-PCR (Retro Transcriptase-Polymerase Chain Reaction) was conducted according to the method of Rodrıguez-Mendoza et al. [81], by using an internal RNA positive control, and confirmed by the methods of Alkowni et al. [15] and Levitzky et al. [21].

### 4.9. Molecular Modeling Studies

#### 4.9.1. Homology Modelling and Protein Preparation

The protein sequences of ToBRFV CP (A0A0S2SZX3 CAPSD_TBRFV) and the template TMV CP (P69687 CAPSD_TMV) were obtained from UniProtKB [83] in FASTA format [123,124] and aligned with BLAST-P. The crystal structure of TMV CP was retrieved from the Protein Data Bank (PDB code: 1ei7) [59]. Homology model for the ToBRFV CP was developed on the TMV CP backbone using Maestro Version 12.6.144 Prime Homology Modeling [88]. Manual editing of the multiple sequence alignments was performed to remove any gap, to modify the 14 different residues, and to align the highly conserved domains. Ramachandran plot calculation [125] was performed for validating the stereochemical quality of the 3D model by PROCHECK [87]. Missing side chains and hydrogens were added, and the resulting structure was optimized using the Protein Preparation Wizard embedded in Schrödinger Suite 2020 [86], pH was set to 6 ± 1 value. Water molecules were removed according to the protocol already described by Sastry et al. [126]. The structure was then energy-minimized within OPLS3 force field [127] to constrain heavy atoms.

#### 4.9.2. Ligands Preparation

The chemical structures of **1**, **2,** and ribavirin were built with Maestro Build Panel [86] and energetically minimized with Ligprep module [128] using OPLS3e force field [127]. For each ligand, conformational search was performed taking into account all possible tautomers and protonation states at a pH of 6 ± 1.0. The ligands set also included the reference ligands ribavirin, already reported in previous works as TMV CP inhibitor [73,89]. The generated conformers were then clustered by means of the Clustering of conformer tool, and the lowest energy conformer from each cluster, for each ligand, was considered for the following docking studies.

#### 4.9.3. Docking Studies

The Receptor Grid Generation tool in the Glide module [129] was used to set up a grid that allowed the prepared ligands to bind into the receptor pocket [126]. A 25 × 25 × 25 Å^3^ grid box was centered at the active site of ToBRFV CP, defined on those residues that are located between two CP subunits—Asn73, Ala74, Val75, Lys134, Thr136, Gly137, Tyr139, Ser143, Lys253, Val255, Val260, and Lys268 [73]. The Glide-SP and the Glide-XP flexible docking approaches were consecutively applied [129]. For each ligand, they resulted in agreement, proposing similar binding modes. The protein-ligand Interaction Profiler tool (https://plip-tool.biotec.tu-dresden.de/plip-web/plip/index, accessed on 20th October 2021) [130] and Ligand interaction diagram Maestro’s tool [86] were used to analyze the target-ligand complex interactions.

### 4.10. Statistical Analysis

The effect of treatments was assessed using the analysis of variance (ANOVA), followed by Tukey HSD test mean separation at *p* ≤ 0.05. Statistics were performed by using the “Statistica” software package, version 8.0, Statsoft Inc. [131].

## 5. Conclusions

ToBRFV is transmitted by contact and can remain infective for months, and control measures are limited and based on the elimination of infected plants and strict hygiene measures [120]. Currently used disinfection systems are often not very effective or potentially dangerous for the operator [8]. Due to the relevance of this virus, new effective, sustainable, and operator safe antiviral agents as well as new formulated products useful for the containment of the pathogen in the field must be developed. Additionally, 4-hydroxybenzoic acid (**2**) was identified as the alkaline autoxidation product of both the methanolic extract of the leaves of *C. micrantum* and of catechinic acid (**1**). Then, **1**, **2**, and AME successfully deactivated viral infectivity of ToBRFV. The loss of virus infectivity and the absence of viral genome in the host plant indicated a possible disassembly of virus CP. Docking simulations highlighted that **1** and **2** bound ToBRFV CP into the active site between the two protein subunits. This suggested that the binding to key residues in the active site may influence the interactions between the two chains, affecting the stability of CP **1** and **2**, and providing a basis for the study of natural compounds targeting ToBRFV CP. To our knowledge, this is the first study of antiviral activity of plant derived compounds against ToBRFV. Moreover, a computational approach on ToBRFV CP is reported here for the first time, and catechinic acid (**1**) revealed the best binding energy value.

In conclusion, the in vitro experiment, combined with inoculation and modelling studies, showed that the virus can be degraded, and therefore, **1** and **2** could be developed as environmental virucides, but their direct use on the host as plant protectors is a promising prospect not yet sufficiently investigated.

## Figures and Tables

**Table 1:** Qualitative evaluation of the presence of ToBRFV during the antiviral activity assay.

Treatments	Concentration (ppm)	Tukey HSD Test ^c^	Inoculation	RT-PCR ^d^		RT-PCR ^e^		RT-PCR ^f^	
				C S	S s	C S	S s	C S	S s
1	32	a	YES	N	N	N	N	N	N
AME	32	a	YES	N	N	N	N	N	N
2	32	a	YES	N	N	N	N	N	N
NaOCl	20,000	a	YES	N	N	N	N	N	N
DW (PC) ^a^	-	b	YES	P	P	P	P	P	P
DW (NC) ^b^	-	a	NO	N	N	N	N	N	N

The test was performed in 3 replicates and 3 different trials. ^a^ DW (PC): distilled water (Positive Control: inoculated and not treated wells); ^b^ DW (NC): distilled water (Negative Control: not inoculated and not treated wells); ^c^ different letters indicate significant difference among treatments (*p* ≤ 0.05, Tukey HSD test); ^d^ method of Rodriguez-Mendoza et al. [81]; ^e^ method of Alkowni et al. [15]; ^f^ method of Levitzky et al. [21]; C S = Cotton swab; S s = Swab solution; N = negative; P = positive.

**Table 2:** Qualitative evaluation of the final viability of the virus.

Treatments	Concentration (ppm)	Tukey HSD Test ^c^	RT-PCR ^d^	RT-PCR ^e^	RT-PCR ^f^
1	32	a	N	N	N
AME	32	a	N	N	N
2	32	a	N	N	N
NaOCl	20,000	a	N	N	N
DW (PC) ^a^	-	b	P	P	P
DW (NC) ^b^	-	a	N	N	N

The test was performed in 3 replicates and 3 different trials. ^a^ DW (PC): distilled water (Positive control: inoculated and not treated wells); ^b^ DW (NC): distilled water (Negative control: not inoculated and not treated wells); ^c^ different letters indicate significant difference among treatments (*p* ≤ 0.05, Tukey HSD test). ^d^ method of Rodriguez-Mendoza et al. [81]; ^e^ method of Alkowni et al. [15]; ^f^ method of Levitzky et al. [21]; N = negative; P = positive.

**Table 3:** Docking interaction parameters of the studied compounds.

Ligand Molecules	Glide Binding Energy (kcal/mol)	H-bond Interacting Amino Acids	Hydrophobic Interactions/π-Cation Interactions	Salt Bridge
4-hydroxybenzoic acid	−25.688	Asp219B, Glu222B, Lys253B, Pro254B, Lys268B	Asp219B, Lys253B, Val255B, Lys268B	Lys268
Catechinic acid	−38.794	Asn73A, Gly137A, Tyr139A, Ser143A, Lys268B	Val260B	-
Ribavirin	−35.285	Asn73A, Lys134A, Gly137A, Leu138A, Ser143A, Pro254B, Lys268B	-	-

## Data Availability

The data presented in this study are available upon request from the corresponding author.

## Supplementary Materials

The following supporting information can be downloaded, Figure S1. Catechinic acid (1) and 4-hydroxybenzoic acid (2); Figure S2. 1H NMR (600 MHz, CD_3_OD) spectrum of 4-hydroxybenzoic acid (2); Figure S3. HSQC (600 MHz, CD_3_OD) spectrum of 4-hydroxybenzoic acid (2); Figure S4. HMBC (600 MHz, CD_3_OD) spectrum of 4-hydroxybenzoic acid (2); Figure S5. ESI(-)-HRMS spectrum of 4-hydroxybenzoic acid; Figure S6. 1H NMR (600 MHz, CD_3_OD) spectrum of catechinic acid (1); Figure S7. HSQC (600 MHz, CD_3_OD) spectrum of catechinic acid (1); Figure S8. HMBC (600 MHz, CD_3_OD) spectrum of catechinic acid (1); Figure S9. COSY (600 MHz, CD_3_OD) spectrum of catechinic acid (1); Figure S10. Experimental set up for antiviral activity assay and plant inoculation; Figure S11. Binding pose (a) and interactions (b) of 4-hydroxybenzoic acid at the ToBRFV CP active site; Figure S12. Binding pose (a) and interactions (b) of ribavirin at the ToBRFV CP active site; Table S1. Preliminary test: qualitative evaluation of RNA degradation of the viral agent after an exposure time of 60 s to different concentrations of catechinic acid (1), 4-hydroxybenzoic acid (2) and AME.

## Sample Availability

Samples of the compounds are available from the authors.
