# Supplementary material for: Autoxidation Products of the Methanolic Extract of the Leaves of Combretum micranthum Exert Antiviral Activity against Tomato Brown Rugose Fruit Virus (ToBRFV)
Source: Molecules. 2022 Jan 24;27(3):760. doi: 10.3390/molecules27030760 (PMC8838289; doi:10.3390/molecules27030760)
Supplement: Supplementary file 1 [file molecules-27-00760-s001.zip › molecules-1521548 (2).pdf]

Supplementary Material for:

Autoxidation products of the methanolic extract of the leaves of *Combretum micranthum* exert antiviral activity against *Tomato brown rugose fruit virus* (ToBRFV)

Valeria Iobbi [1*], Anna Paola Lanteri [2*], Andrea Minuto [2], Valentina Santoro [4], Giuseppe Ferrea [3], Paola Fossa [1], Angela Bisio [1,]**

[1] Department of Pharmacy, University of Genova, Viale Cembrano 4, 16148 Genova, Italy; bisio@difar.unige.it ; valeria.iobbi@edu.unige.it; paola.fossa@unige.it
[2] CeRSAA Centro di Sperimentazione e Assistenza Agricola, Regione Rollo 98, 17031 Albenga, Italy; labfito@cersaa.it; andrea.minuto@rivlig.camcom.it
[3] Azienda Sanitaria Locale 1. Regione Liguria, Via Aurelia 97, 18038 Bussana, Italy;
[4] Department of Pharmacy, University of Salerno, Via Giovanni Paolo II 132, 84084 Salerno, Italy; vsantoro@unisa.it
* Equally contributed
** Correspondence: bisio@difar.unige.it

CONTENT

Figure S1. Catechinic acid (**1**) and 4-hydroxybenzoic acid (**2**)
Figure S2. ^{1}H NMR (600 MHz, CD_3OD) spectrum of 4-hydroxybenzoic acid (**2**).
Figure S3. HSQC (600 MHz, CD_3OD) spectrum of 4-hydroxybenzoic acid (**2**).
Figure S4. HMBC (600 MHz, CD_3OD) spectrum of 4-hydroxybenzoic acid (**2**).
Figure S5. ESI(-)-HRMS spectrum of 4-hydroxybenzoic acid
Figure S6. ^{1}H NMR (600 MHz, CD_3OD) spectrum of catechinic acid (**1**).
Figure S7. HSQC (600 MHz, CD_3OD) spectrum of catechinic acid (**1**).
Figure S8. HMBC (600 MHz, CD_3OD) spectrum of catechinic acid (**1**).
Figure S9. COSY (600 MHz, CD_3OD) spectrum of catechinic acid (**1**).
Figure S10. Experimental set up for antiviral activity assay and plant inoculation.
Figure S11. Binding pose (a) and interactions (b) of 4-hydroxybenzoic acid at the the ToBRFV CP active site.
Figure S12. Binding pose (a) and interactions (b) of ribavirin at the ToBRFV CP active site.
Table S1. Preliminary test: qualitative evaluation of RNA degradation of the viral agent after an exposure time of 60 seconds to different concentrations of catechinic acid (**1**), 4-hydroxybenzoic acid (**2**) and AME. 9

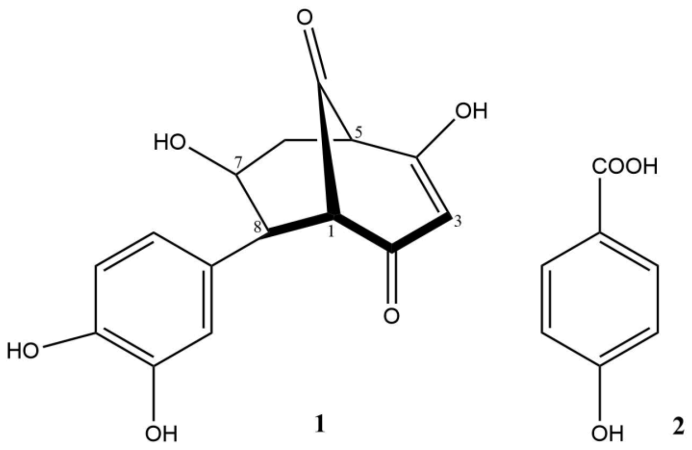

Figure S1. Catechinic acid (**1**) and 4-hydroxybenzoic acid (**2**)

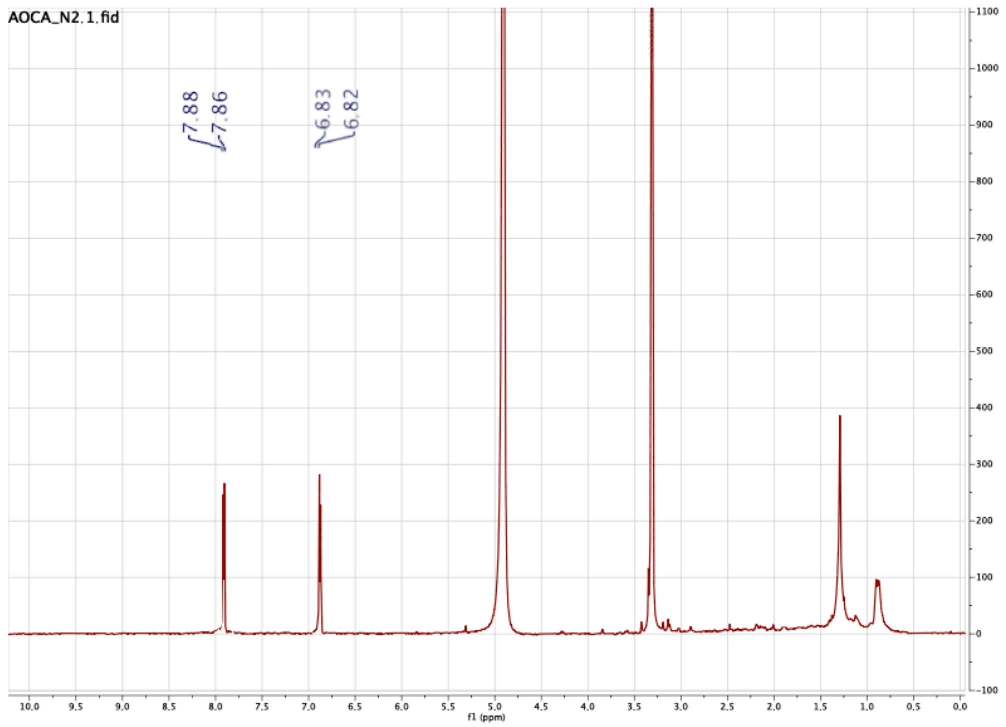

Figure S2. ^{1}H NMR (600 MHz, CD_3OD) spectrum of 4-hydroxybenzoic acid (**2**).

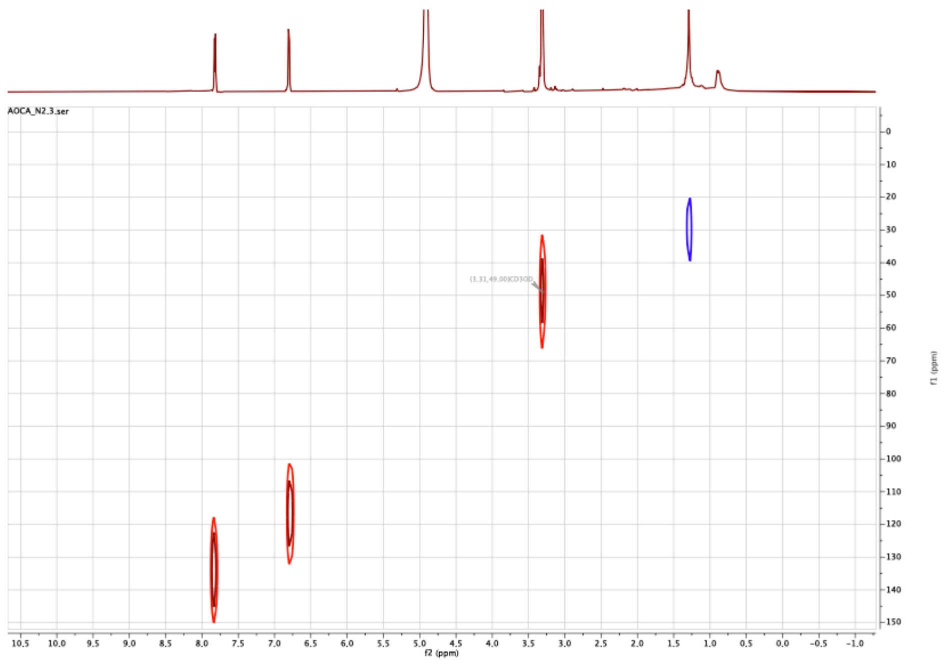

Figure S3. HSQC (600 MHz, CD_3OD) spectrum of 4-hydroxybenzoic acid (**2**).

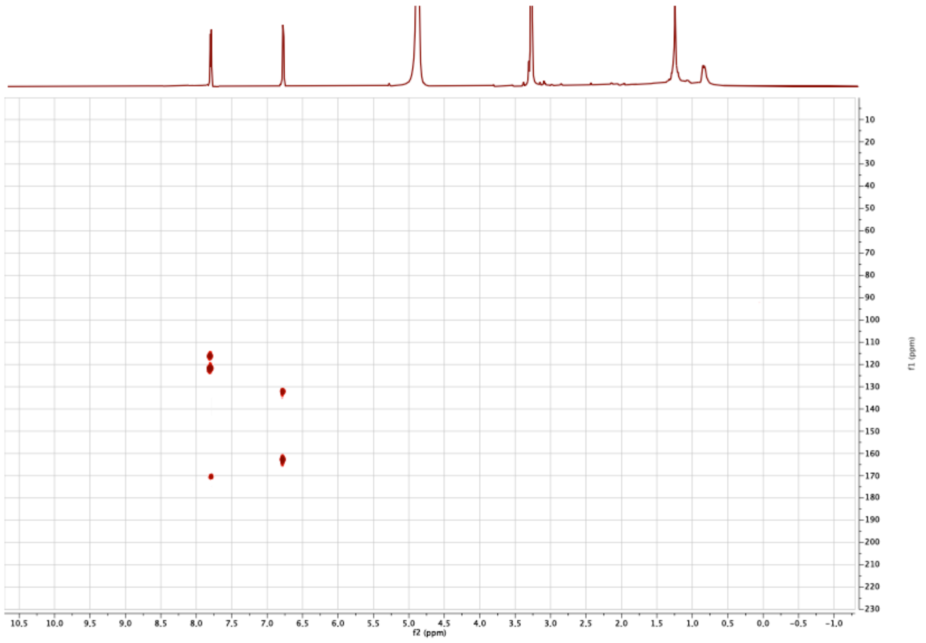

Figure S4. HMBC (600 MHz, CD_3OD) spectrum of 4-hydroxybenzoic acid (**2**).

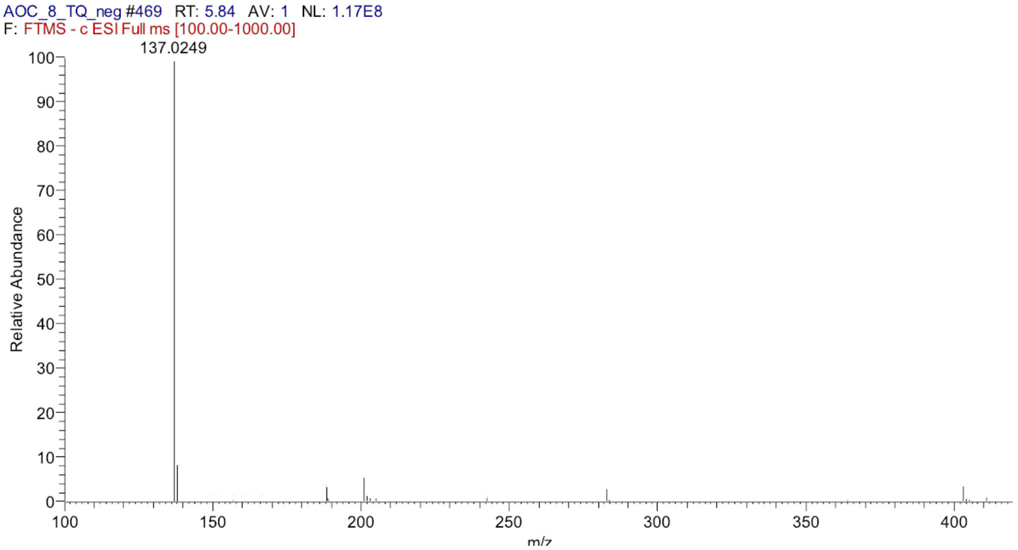

Figure S5. ESI(-)-HRMS spectrum of 4-hydroxybenzoic acid

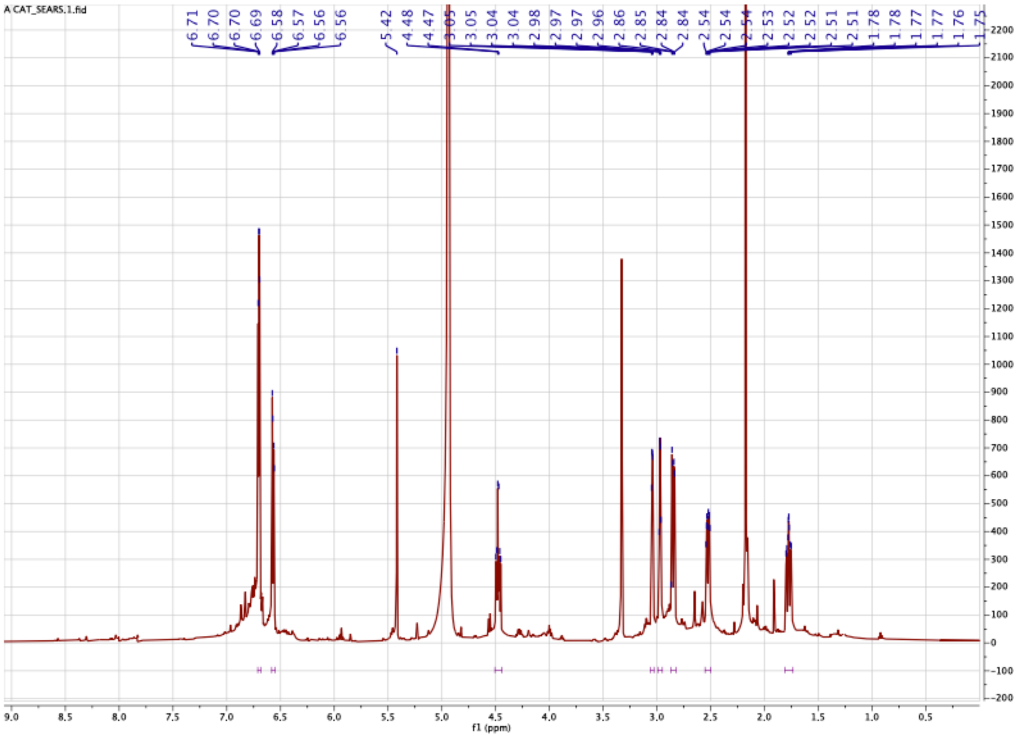

Figure S6. ¹H NMR (600 MHz, CD_3OD) spectrum of catechinic acid (**1**).

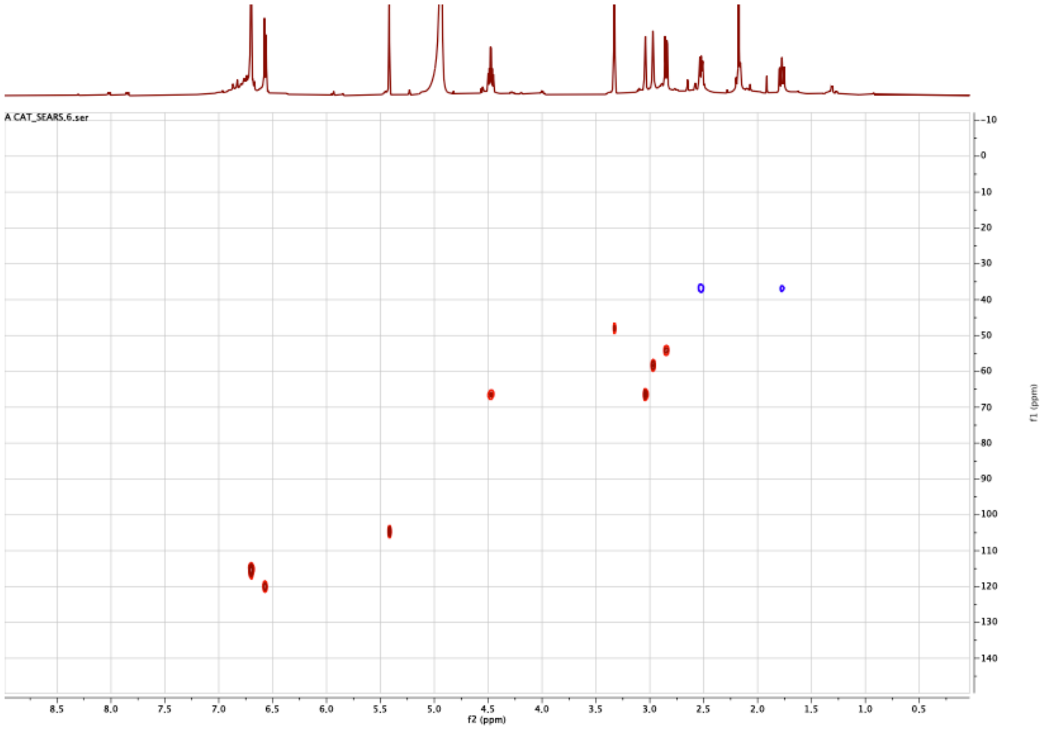

Figure S7. HSQC (600 MHz, CD_3OD) spectrum of catechinic acid (**1**).

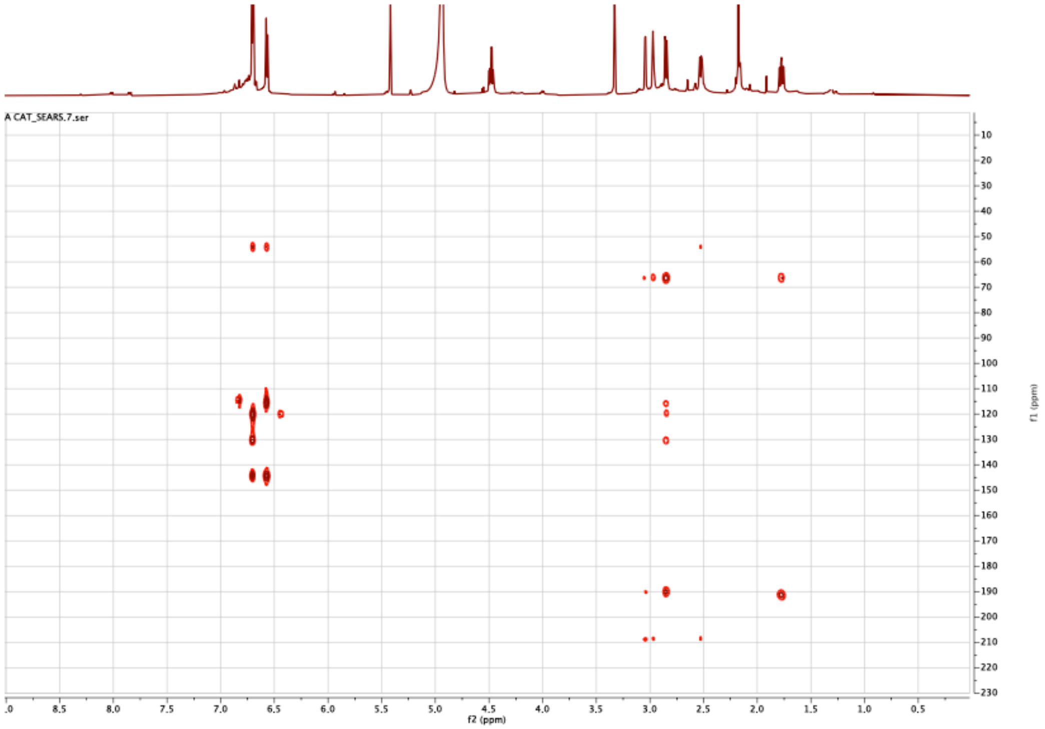

Figure S8. HMBC (600 MHz, CD_3OD) spectrum of catechinic acid (**1**).

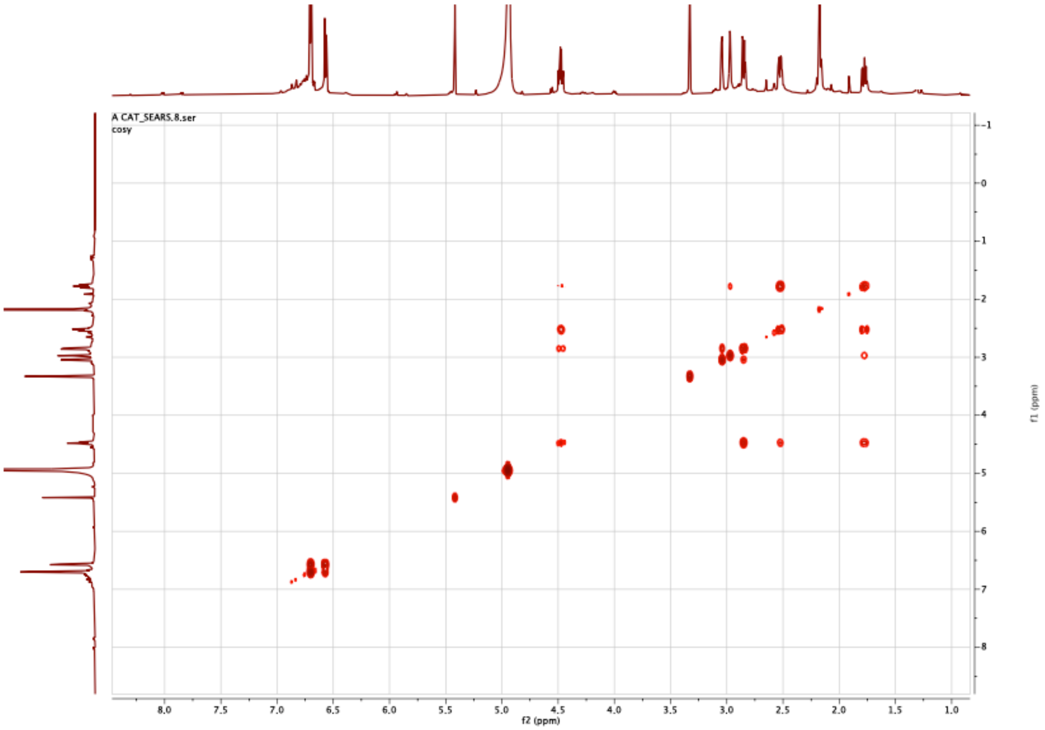

Figure S9. COSY (600 MHz, CD_3OD) spectrum of catechinic acid (**1**).

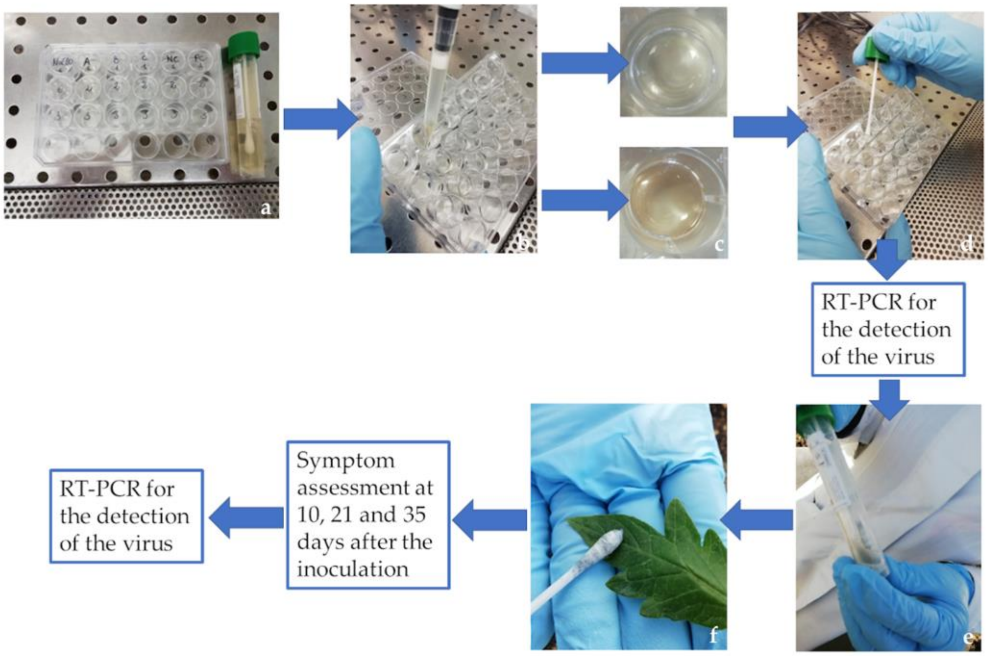

Figure S10. Experimental set up for antiviral activity assay and plant inoculation.
a: cell culture multiwell plate and swab used for the *in vitro* assay; b: inoculation of the flat bottom well surface; c: not treated and treated well; d: sampling of the flat bottom well surface; e: swab with carborundum used for the plant inoculation; f: plant inoculation with cotton swab

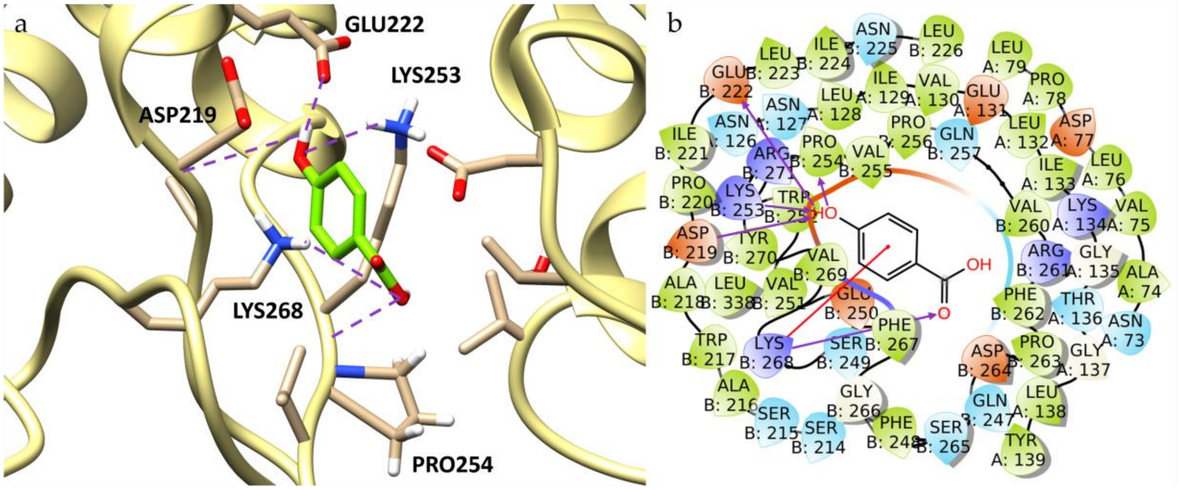

Figure S11. Binding pose (a) and interactions (b) of 4-hydroxybenzoic acid at the the ToBRFV CP active site.

(a): the protein is reported as light yellow ribbons, 4-hydroxybenzoic acid is reported as lime green capped sticks. H-bonds are presented as purple dotted lines. (b): 4-hydroxybenzoic acid is surrounded by the protein residues represented as follows: the negatively charged residues are indicated in red, polar residues are in cyan, hydrophobic residues are shown in green. H-bonds are depicted as purple arrows; π-cation interactions are reported as red lines.

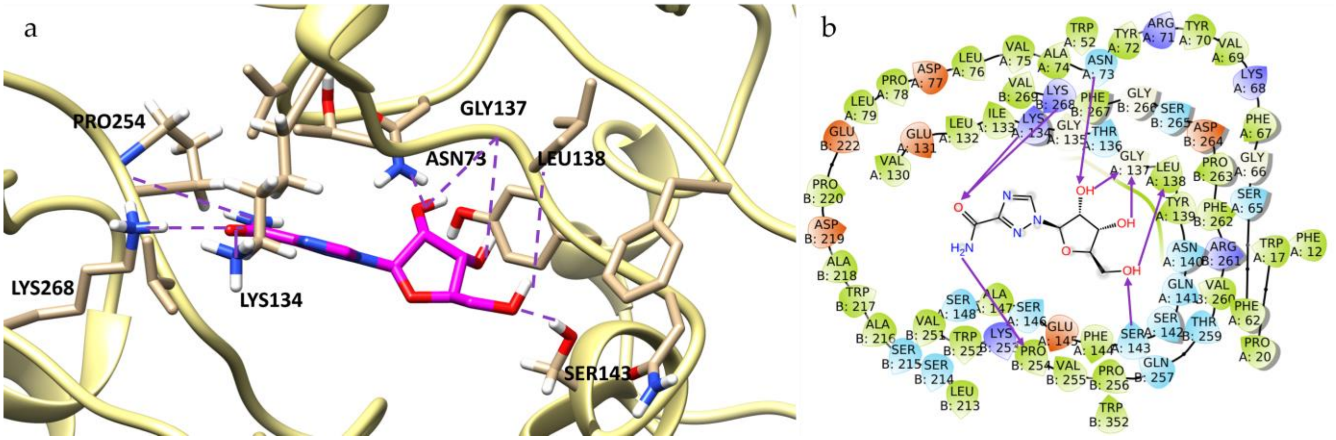

Figure S12. Binding pose (a) and interactions (b) of ribavirin at the ToBRFV CP active site.

(a): the protein is reported as light yellow ribbons, ribavirin is reported as magenta capped sticks. H-bonds are presented as purple dotted lines. (b): ribavirin is surrounded by the protein residues represented as follows: the negatively charged residues are indicated in red, polar residues are in cyan, hydrophobic residues are shown in green. H-bonds are depicted as purple arrows.

Table S1. Preliminary test: qualitative evaluation of RNA degradation of the viral agent after an exposure time of 60 seconds to different concentrations of catechinic acid (**1**), 4-hydroxybenzoic acid (**2**) and AME.

Treatments	Concentration (ppm)	Tukey HSD test[c]	Inoculation	RT- PCR[d]		RT- PCR[e]		RT- PCR[f]	
				C S	S s	C S	S s	C S	S s
**1**	4000	a	NO	N	N	N	N	N	N
**1**	800	a	NO	N	N	N	N	N	N
**1**	160	a	NO	N	N	N	N	N	N
**1**	32	a	NO	N	N	N	N	N	N
**1**	6.4	b	NO	P	P	P	P	P	P
AME	4000	a	NO	N	N	N	N	N	N
AME	800	a	NO	N	N	N	N	N	N
AME	160	a	NO	N	N	N	N	N	N
AME	32	a	NO	N	N	N	N	N	N
AME	6.4	b	NO	P	P	P	P	P	P
**2**	4000	a	NO	N	N	N	N	N	N
**2**	800	a	NO	N	N	N	N	N	N
**2**	160	a	NO	N	N	N	N	N	N
**2**	32	a	NO	N	N	N	N	N	N
**2**	6.4	b	NO	P	P	P	P	P	P
NaOCl	20000	a	NO	N	N	N	N	N	N
DW (PC)[a]	-	b	NO	P	P	P	P	P	P
DW (NC)[b]	-	a	NO	N	N	N	N	N	N

The test was performed in 3 replicates. [a] DW (PC): Distilled water (Positive Control: inoculated and not treated wells); [b]DW (NC): Distilled water (Negative Control: not inoculated and not treated wells); [c] Different letters indicate significant difference among treatments ($p \leq 0.05$, Tukey HSD test); [d] method of Rodriguez-Mendoza et al. [1]; [e] method of Alkowni et al. [2]; [f] method of Levitzky et al. [3]; C S = Cotton swab; S s = Swab solution; N = negative; P = positive.

References

1. Rodríguez-Mendoza, J.; García-Ávila, C.d.J.; López-Buenfil, J.A.; Araujo-Ruiz, K.; Quezada-Salinas, A.; Cambrón-Crisantos, J.M.; Ochoa-Martínez, D.L. Identificación de *Tomato brown rugose fruit virus* por RT-PCR de una región codificante de la replicasa (RdRP). *Rev. Mex. Fitopatol.* **2019**, *37*, 345-356.
2. Alkowni, R.; Alabdallah, O.; Fadda, Z. Molecular identification of *Tomato brown rugose fruit* virus in tomato in Palestine. *J. Plant Pathol.* **2019**, *101*, 719-723, doi:10.1007/s42161-019-00240-7.
3. Levitzky, N.; Smith, E.; Lachman, O.; Luria, N.; Mizrahi, Y.; Bakelman, H.; Sela, N.; Laskar, O.; Milrot, E.; Dombrovsky, A. The bumblebee *Bombus terrestris* carries a primary inoculum of *Tomato brown rugose fruit* virus contributing to disease spread in tomatoes. *PLoS One* **2019**, *14*, e0210871, doi:10.1371/journal.pone.0210871.
